# Assessment of cybersickness induced by augmented reality ultrasound procedures using head-mounted display

**DOI:** 10.3389/fdgth.2026.1804183

**Published:** 2026-05-19

**Authors:** Thomas Saliba, Pierre Frossard, Giuseppe Gullo

**Affiliations:** 1 Faculty of Medicine and Pharmacy, Vrije Universiteit van Brussel (VUB), Brussels, Belgium; 2 Radiology Department, Centre Hospitalier Universitaire Vaudois (CHUV), Lausanne, Switzerland

**Keywords:** augmented reality, cybersickness, diagnostic, interventional, needle, ultrasound

## Abstract

**Introduction:**

Augmented Reality (AR) has emerging applications in medical imaging, particularly ultrasound. However, concerns about cybersickness may hinder widespread adoption. This is the first study to evaluate cybersickness symptoms experienced by healthcare professionals using a commercially available AR head-mounted display (HMD) during simulated abdominal ultrasound and vascular access procedures.

**Methods:**

Fifty-two radiologists, sonographers, and CT technologists performed either an abdominal ultrasound (*n* = 29) or a venous cannulation (*n* = 23) on phantoms using an augmented reality headset paired with an ultrasound probe. Participants received minimal training and were evaluated using the Simulator Sickness Questionnaire (SSQ), which quantifies nausea (N), oculomotor disturbance (O), and disorientation (D). Task completion times and first-pass success rates (for vascular access) were recorded. Statistical analysis included Mann–Whitney U and Spearman's correlation tests.

**Results:**

Median task completion times were 11.83 min for abdominal ultrasound and 7 min for venous cannulation, with an 87% first-pass success rate in the latter. Median SSQ total scores remained below the threshold of concern [abdominal: 15 (N = 10, O = 15, D = 14); cannulation: 11 (N = 0, O = 15, D = 14)]. Pooled scores followed the pattern O > D > N (N = 5, O = 15, D = 14), consistent with previous AR research. No significant differences in SSQ scores were found between groups, though small effects cannot be formally ruled out due to a weak experimental power. A weak positive correlation (r = 0.27) existed between task time and SSQ scores.

**Discussion:**

AR-guided ultrasound using a commercially available HMD is feasible and well-tolerated by healthcare professionals, with minimal cybersickness irrespective of age or sex. The setup demonstrated ease of adoption and may theoretically offer ergonomic and workflow advantages in clinical settings.

## Introduction

The use of virtual reality (VR), defined as an artificial environment which is experienced through sensory stimuli (such as sights and sounds) provided by a computer and in which one's actions partially determine what happens in the environment and augmented reality (AR), defined as an enhanced version of reality created by the use of technology to overlay digital information on an image of something being viewed through a device, in medical fields is on the rise, with various applications, ranging from therapeutics to helping prepare patients for MRIs (1–5). As the technology 's possible use-cases grow in the field of radiology, the possible ways of integrating AR headsets into the field of ultrasound are increasing as the technology begins to mature and become more accessible.

A common issue faced by sonographers and vascular technologists is musculoskeletal discomfort, with up to 90% of respondents saying that they scanned patients whilst in physical pain due to their scanning positions (6). Up to 40% of respondents indicated suffering from neck pain, with similar numbers suggesting that the lower back was also affected (6). Therefore, it is possible that this may also be an area in which AR with virtual controls could have an impact by reducing the burden placed on sonographers by having to adopt awkward positions to image the patients and see their screen, all the while maintaining a position that gives them access to the ultrasound machine settings (7).

A large body of work has evaluated VR/AR-based training tools for teaching procedural and diagnostic ultrasound skills, demonstrating benefits in engagement, skill acquisition, and simulation fidelity (8–12). Image-guided procedures have also been researched, with AR overlays having been explored for vascular access, needle guidance, and other point-of-care procedures, showing potential for enhancing accuracy and reducing cognitive load (13–16).

Although the potential applications of augmented reality (AR) are extensive, its widespread adoption may be hindered by significant limitations arising from the multifaceted nature of its associated side effects (17). Cybersickness is widely documented in VR and AR, which may cause concerns as to the ability to apply the technology in real world settings (18, 19). In VR, cybersickness is mainly driven by symptoms of disorientation and nausea, caused by conflicting vestibular and visual inputs (20). However, the causes for cybersickness in VR have been shown to differ from those that cause them in AR, where research has highlighted that eye-strain and oculomotor symptoms may be a primary driver of cybersickness (20). This is particularly relevant for radiological use of AR technology in which constant vision and attentive observation are essential (20). Furthermore, it is well known that AR has the potential to cause cybersickness in users, with some authors claiming that as time spent using AR increases that cybersickness will become an inevitability (21). However, other authors have claimed that cybersickness symptoms are minimal when using AR to learn tasks, leaving much debate as to whether AR can be used without significant side effects (22).

To date, no study has specifically examined the potential effects of cybersickness on conducting ultrasound-based examinations or procedures in conjunction with AR. We conducted a PubMed search {query: “Ultrasonography” [MeSH Terms] AND (“Motion Sickness” [MeSH Terms] OR “Cybersickness” [All Fields])} finding only 5 uncorrelated results. However, proving that it is possible for users to conduct procedures and diagnostic exams using AR without significant side effects is fundamental before any forms of real-world adoption can take place. In this study, we aimed to evaluate the cyber-discomfort experienced by healthcare professionals during AR-guided ultrasound procedures on phantoms (venipuncture and abdominal examination) using an augmented reality head-mounted display (HMD), with SSQ scale ratings as our primary outcome. This is the first exploratory study to evaluate cybersickness, a prominent concern highlighted by previous studies, with a consumer pass-through AR headset (Quest 3), a relatively low-cost and accessible device, for ultrasound tasks.

## Methods

### Participants

We recruited participants who were either radiologists, sonographers or CT technicians with experience using ultrasound to place IV catheters. In order to have an accurate representation of the department's staff, no limits were placed on the participants' age or level of experience. None of the participants had significant exposure to VR or AR, and none had ever tested our setup before the experiment, which was based on previous methodology first presented by Saliba et al. (23)

### Material

We used the Lumify Linear (L12-4) and Convex (C 5-2) probes (Phillips, Amsterdam, Netherlands), which we connected by USB-C to the HMD.

The HMD we used was a Meta Quest 3 (Meta, Menlo Park, California, USA), with no substantial modifications, onto which the Lumify app used by the ultrasound probes was installed. We used the Quest 3's ability to load standard Android apps via a process called sideloading. The apps can then be manipulated by hand gestures, using the Android app that is normally installed on a tablet or phone, and then used in conjunction with the probes. The headset features a 2064 × 2208 per-eye resolution, a 120 Hz refresh rate (which was the setting used during the experiment), a 110° horizontal and 96° vertical rendered field of view (80° binocular overlap, weighs 515 grams, supports an interpupillary distance range of 58–71 mm, and offers a peak angular pixel density of 25 pixels per degree at the center of the visual field (24).

In this setup, the AR ultrasound is not merely mirrored to the HMD; instead, the application runs directly on the HMD, with the probe connected to it via cable. Compared with other *in situ* models, this approach offers two key advantages: much lower cost and elimination of latency that can reach 50 ms for *in situ* US (25, 26).

Video see-through (VST) systems (e.g., Meta 3) are generally more commercially viable and less expensive than optical see-through (OST) systems in radiology, as OST systems are technologically more complex and more sensitive to lighting conditions (25).

### Questionnaire

In order to rate the participants' cybersickness we used the SSQ scale developed by Kennedy et al. that contains 16 items that address the different physical symptoms associated with cybersickness (27). This scale divides symptoms into Nauseaa (N), Oculomotor disturbance (O), and Disorientation (D) (28, 29). Each subscale score is calculated by summing the ratings of the relevant symptoms and applying a category-specific weighting factor (respectively N*9.54, O*7.58, D*13.92. A total SSQ score (TS) can also be derived the same way, applying one more specific factor (TS*3.74) (27).

Scores can be interpreted as follows: negligible (<5), minimal (5–10), significant (10–15), and concerning (15–20). Score exceeding 20 denotes a bad simulator (30). No SSQ rating was performed pre-simulation as this was not deemed necessary. Participants were orally asked if they were in their “usual state of fitness”, with a negative response resulting in exclusion, in line with the original protocol developed by Kennedy et al. (27)

### Overall design

**Figure 1:**
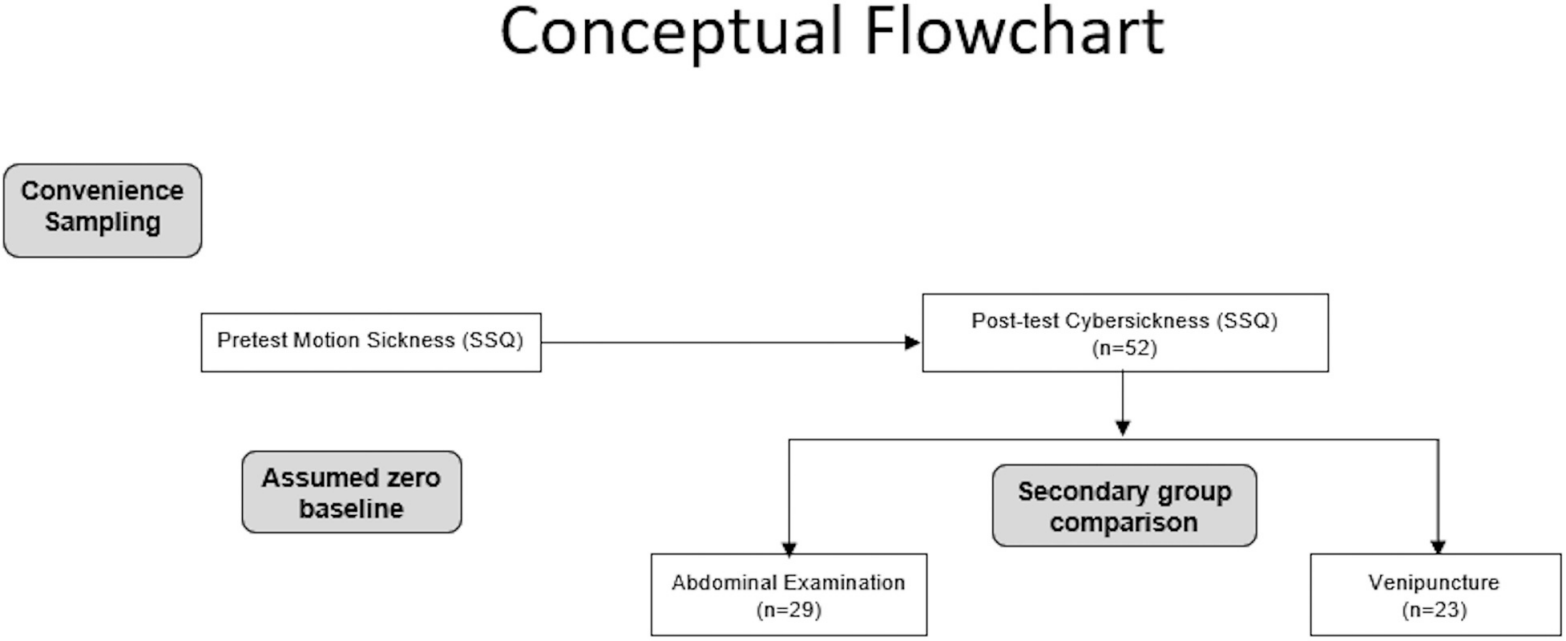
Conceptual flowchart of the experimental procedure.

This study employed a quasi-experimental, posttest-only design. Although a pretest–posttest framework was initially considered, no pretest was conducted, as baseline SSQ scores were assumed rather than empirically measured. This assumption was based on two considerations: (1) its consistency with the methodological approach originally proposed by Kennedy, and (2) the general consensus that a single, standard ultrasound examination is not associated with an increased risk of discomfort, including symptoms resembling motion sickness (Figure 1).

Allocation was based on prior experience. Operators with experience in both categories were assigned to abdominal procedures, while those with experience in only one category were allocated accordingly. This was done to avoid having participants that were not familiar with a certain procedure being asked to do so, which would have added an additional variable All procedures took place under fully illuminated conditions (>500 lux). Participants were allowed to adjust the interpupillary distance of the headset if they wished to do so before the exam, in order to increase their visual comfort.

**Figure 2:**
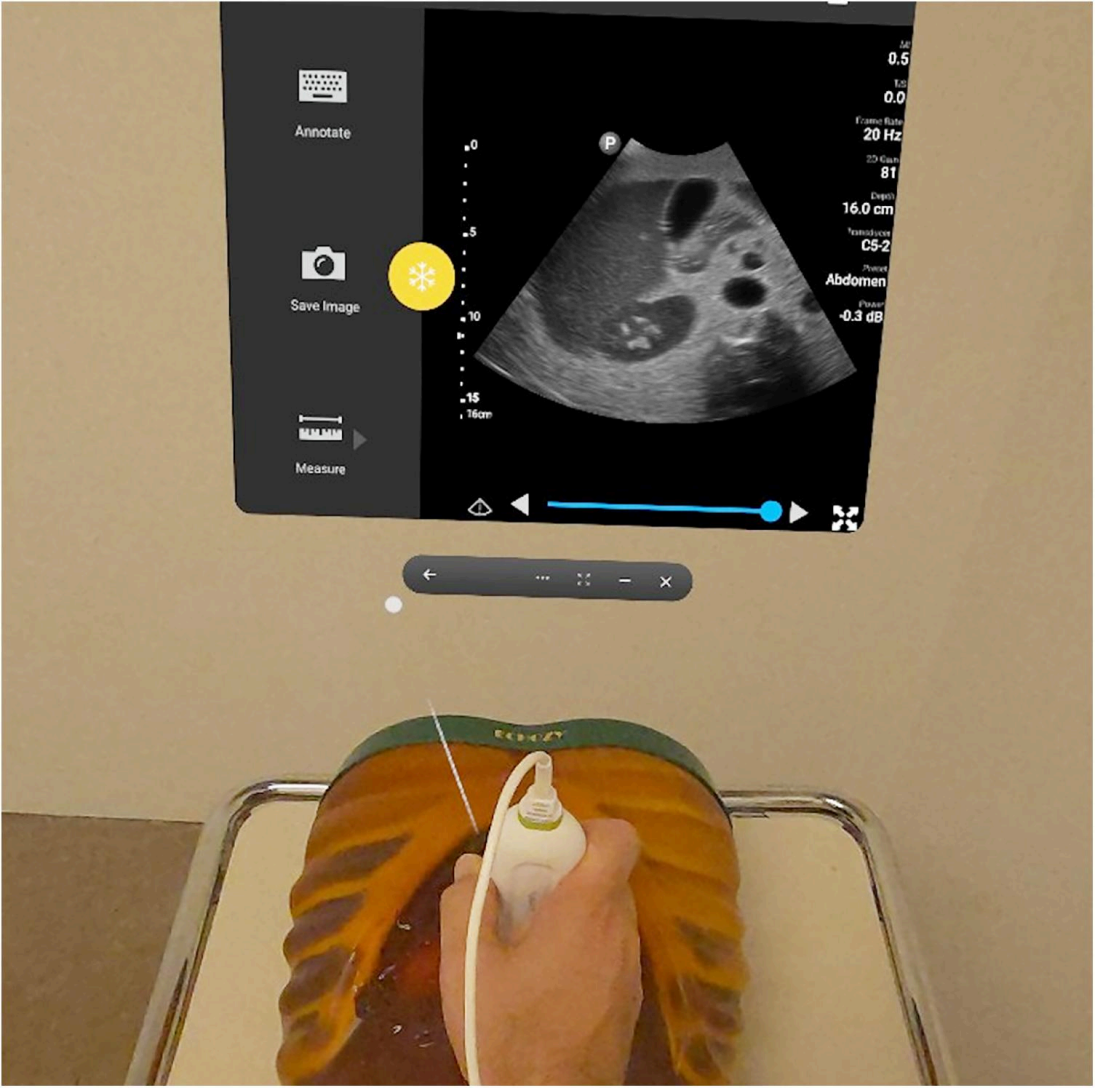
Point of view of the sonographer whilst performing the augmented reality scan on the abdomen phantom.

TasksOne group of participants was required to complete a standard abdominal ultrasound exam on dedicated phantom (Ultrasound Examination Training Phantom “ECHOZY”, Fushimi-ku, Kyoto, Japan) (18) (Figure 2). The participant was seated in the same posture used for an abdominal ultrasound, with the phantom placed laterally on the right side of the participant on the patient table. The time taken to perform the task was recorded and the subjects were asked to rate their cybersickness (SSQ questionnaire) immediately after they had removed the AR headset. SSQ was self-administered.

**Figure 3:**
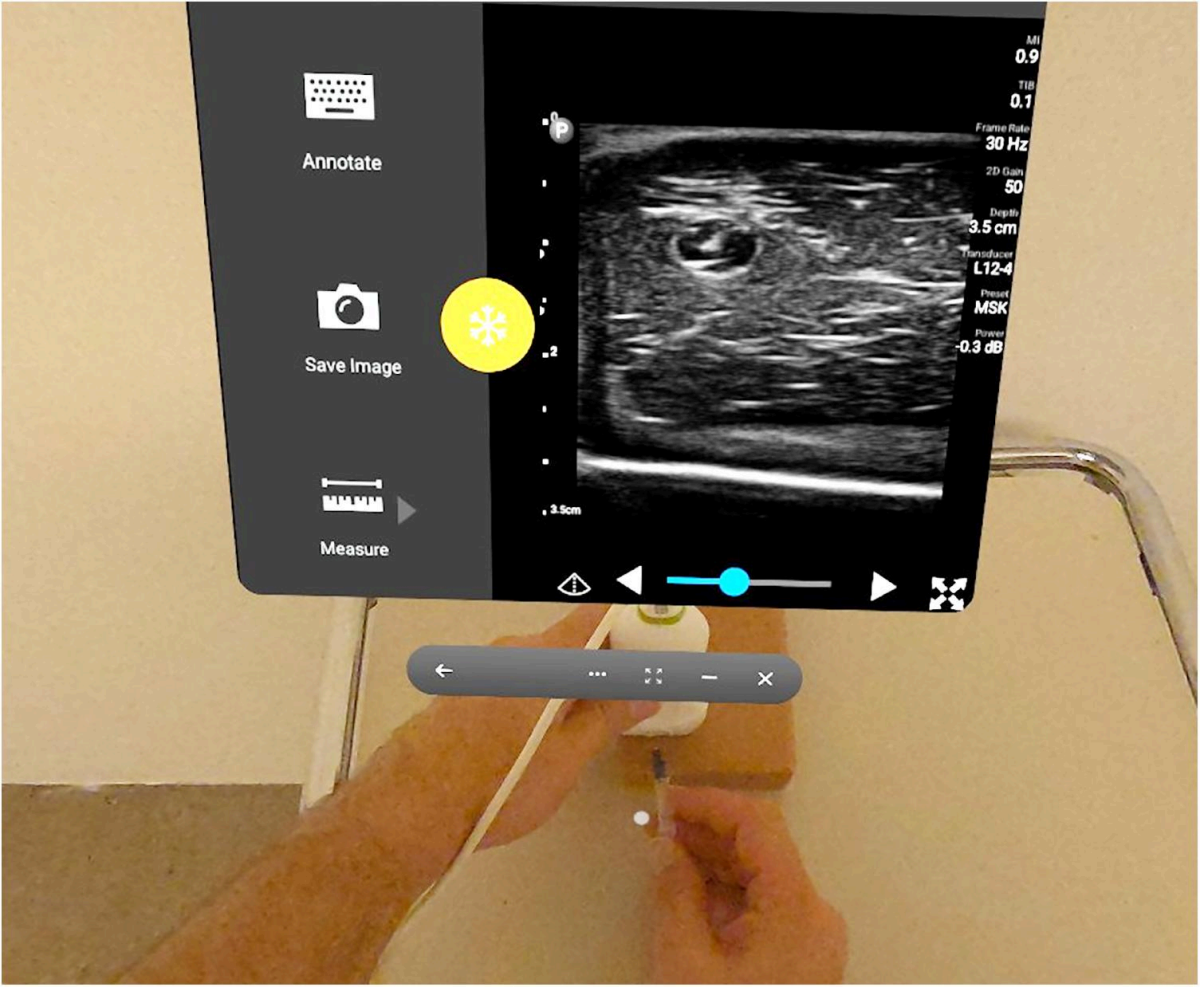
Point of view of the sonographer whilst performing the augmented reality scan on the vascular access phantom.

The second group was required to perform a venous cannulation on another dedicated phantom [Vascular access training phantom, Computerized Imaging Reference Systems (CIRS) Model 072, Norfolk, VA, USA] (Figure 3). The participant faced the phantom placed on a patient table as it more closely reflects the position usually used for venipunctures. Cannulation was performed out of-plane following the technique described by Donaldson, Morello, Junewick, O'Donovan, and Lim-Dunham (31). The time taken to perform the task and whether they were successful on their first attempt was recorded. The SSQ questionnaire was applied the same way as for group 1.

### Statistics

Statistics were performed using STATA (Version 18.0, StataCorp, TX, USA). The sample size was not estimated *a priori*; it was determined based on the availability of healthcare professionals at the time of the study (convenience sampling).

Comparisons were performed using a t-test or, if normality was not met, a Mann–Whitney test, after verifying normality with the Shapiro–Wilk test. The significance threshold was set at 0.05. Pearson's correlation (or Spearman's rho for non-normally distributed variables) was used to assess the relationship between cybersickness and participant characteristics This is interpreted as follows; 0.10–0.39 Weak correlation 0.40–0.69 Moderate correlation >0.70 Strong correlation (32).

### Ethics

We sought clarification on the need for Ethics Committee (EC) approval of the CER-VD (ethics committee of the canton of Vaud, Switzerland). The study, registered as “Req-2025-00332,” was deemed outside the EC's scope and did not require formal authorization.

## Results

We recruited 52 participants who were either radiologists, sonographers or CT technicians with experience using ultrasound to place IV catheters. They were split into two groups, with 29 performing abdominal ultrasounds and 23 performing cannulations (Figure 1).

There were no significant differences in sex or age between the groups (Table 1).

**Table 1:** Comparison of patients’ characteristics.

Characteristic	All (*n* = 52)	Abdominal US (*n* = 29)	Venipuncture (*n* = 23)	*P*-value
Sex (Male, %)	30 (58%)	17 (59%)	13 (57%)	1.00
Age (years)	35 (24–63)	38 (26–63)	34 (24–49)	0.08

We assessed normality using the Shapiro–Wilk test, which indicated a deviation from normality. Consequently, median values with interquartile ranges (IQR) were reported, as they better represent the central tendency for right-skewed, non-mesokurtic data.

None of the participants suffered any side effects that required them to stop the experiment, and all the subjects successfully completed their tasks. Pooled scores were N = 5, O = 15, D = 14, TS = 13 (Table 2). Assuming a zero baseline for the pretest SSQ score, the pretest–posttest comparison would indicate a statistically significant difference (*p* < 0.001) (Table 2).

**Table 2:** Augmented reality data and simulator sickness questionnaire (SSQ) score.

Characteristic	All (*n* = 52)	Abdominal US (*n* = 29)	Venipuncture (*n* = 23)	*P*-value	Effect size Vargha and Delaney's A (A12)
Time	8.96 (4.17–21.66)	11.83 (5.16–21.66)	7 (4.17–11.33)	<0.001	0.921
1st try puncture (%)	NA	NA	87	NA	NA
N	4.77 (0–38.16)	9.54 (0–38.16)	0 (0)	0.10	0.625
O	15.16 (0–75.8)	15.16 (0–75.8)	15.16 (0–60.64)	0.39	0.569
D	13.92 (0–111.36)	13.92 (0–111.36)	13.92 (0–69.6)	0.77	0.523
TS	13.09 (0–67.32)	14.96 (0–67.32)	11.22 (0–52.36)	0.39	0.570

Unless specified, quantitative variables are expressed as median. Numbers in brackets are ranges.

SSQ Score for Nausea (N), Oculomotor disturbance (O), Disorientation (D), and Total score (TS).

**Figure 4:**
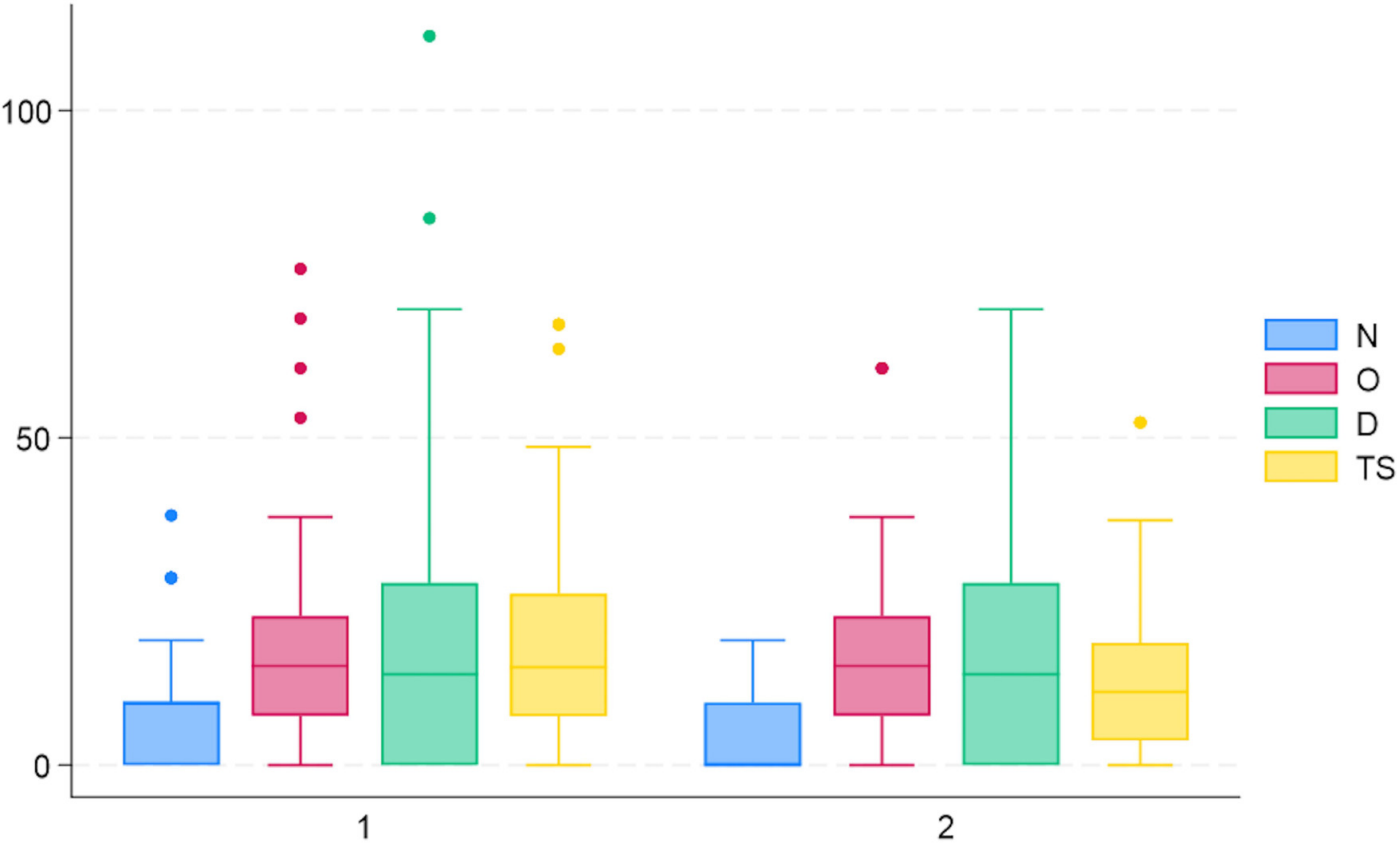
Whisker plot of the total scores given by the abdominal ultrasound group (1) and the venipuncture group (2). The blue box represents nausea (N), the red box represents oculomotor disturbance (O), and the green box represents disorientation (D). The yellow box is the total score (TS).

No significant correlations were found between age, or gender and cybersickness TS (Table 3). A weak correlation (0.27) was found between the time taken to complete the task and cybersickness TS (Table 3, Figure 4)

**Table 3:** Correlation with simulator sickness questionnaire total score.

Characteristic	Correlation (r)	*P*-value
Age	−0.03	0.86
Gender	0.02	0.90
Time	0.27	0.05

Participants tasked with completing an abdominal ultrasound using the AR headset did this in 11.83 min on median. They did not report any concerning discomfort due to cybersickness. N = 10, O = 15, D = 14, TS = 15 (Table 2).

Participants tasked with performing a cannulation using the AR headset did this in 7 min on median, with 87% performing a successful cannulation on the first attempt. They did not report any concerning discomfort due to cybersickness. N = 0, O = 15, D = 14, TS = 11 (Table 2).

**Figure 5:**
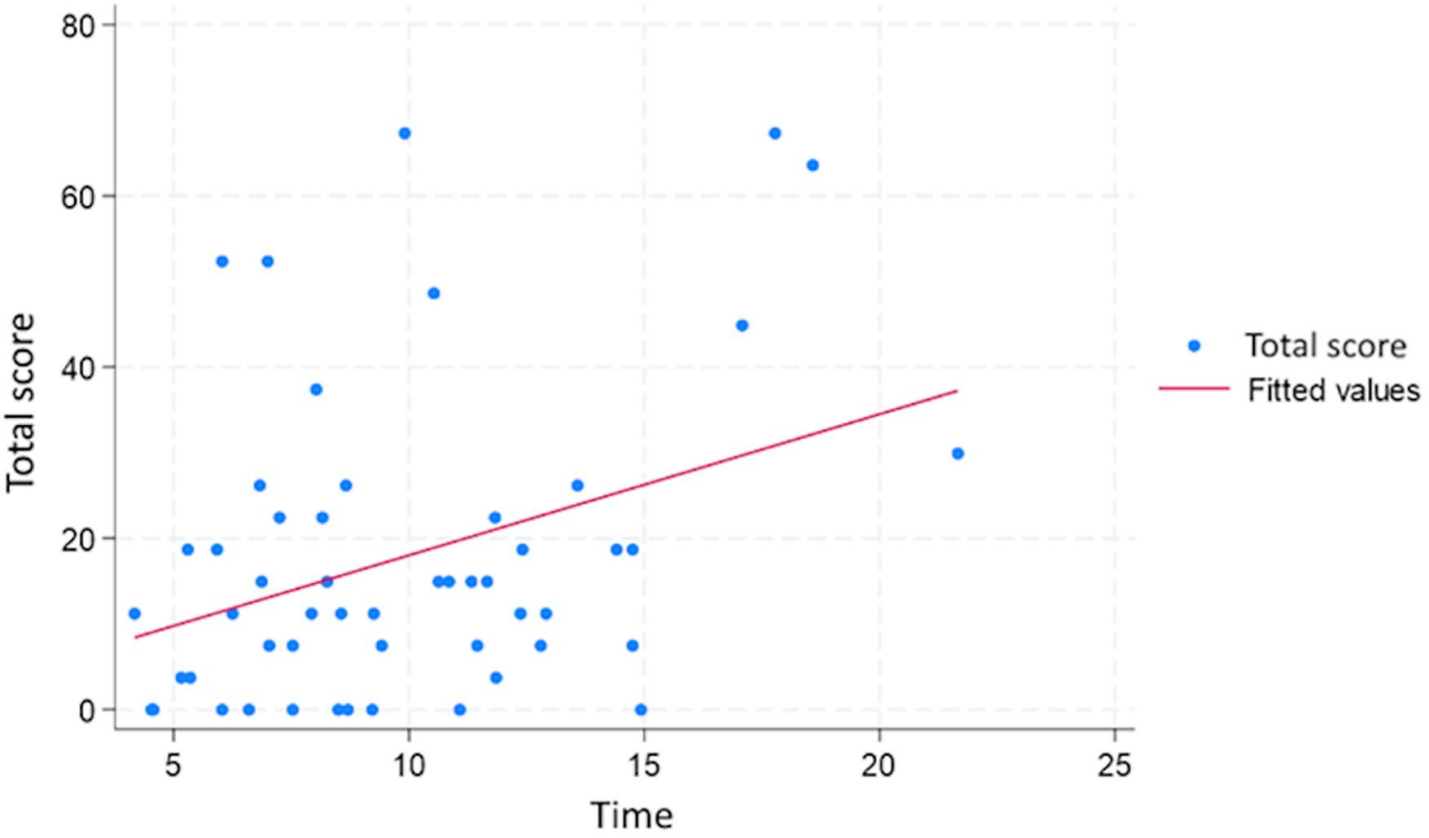
Scatter plot of the time total score of each user against the time taken to perform the exam, showing a weak correlation.

There were no significant differences in the cybersickness between the two groups (Figure 5), though there was a difference in the time taken to complete the tasks, which can be explained by the tasks being different (Table 2).

The absence of difference between the SSQ groups made us perform a *post-hoc* power estimate. *post-hoc* power was estimated by converting the Vargha–Delaney A statistic (Table 2) to Cohen's d, computing the corresponding noncentrality parameter based on group sample sizes, and applying a normal approximation for a two-tailed test at *α* = 0.05. Globally for TS due to the sample size there was only a power of 0.14 to confirm a small effect (Vargha–Delaney A = 0.57), which may suggest that the study was under-powered and may thus miss small but significant effects (33).

## Discussion

There is no universally accepted theory for why cybersickness occurs, though some have emitted hypotheses that it may be due to sensory conflicts, postural instability, eye movement related or evolutionary in nature (19, 34, 35). Although less studied than in VR, AR has been shown to induce cybersickness, generally following the a Disorientation > Nausea > Oculomotor pattern when assessed using the SSQ scale (28). Small-scale research on cybersickness in AR applications, however, have shown a Oculomotor > Disorientation > Nausea pattern to be dominant (28). In our research, both groups had a Oculomotor > Disorientation > Nausea, fitting with previous research.

Visual-vestibular mismatch is another way in which VR has been known to cause cybersickness, wherein there is a conflict between what the subject is seeing and what their inner ear is feeling (36). This theory is supported by the fact that patients with dysfunctional inner ears have increased symptoms, whilst those with non-functional vestibular systems experience no symptoms of cybersickness (37). These problems are to be expected when vection, which is the perception of self-motion due to visual cues, conflicts with the vestibular system, causing nausea (36). Recent studies have shown that AR is less subject to these problems as the user maintains visual cues due to seeing the images super-imposed on the real background which acts as a visual anchor (28). However, a study found that around 10% of users are subject to severe symptoms which can lead to aborting the use of AR, though none of our subjects experienced such symptoms (28). Low symptom severity may be due to the static nature of the AR display and absence of perceptual lag.

The postural instability theory suggests that situations associated with motion sickness are characterized by a period where patterns of movement control are changed to adapt to a new stimulus (37).This adaptation results in less efficient movements and therefore postural instability (37). In these cases, visual stimulus also plays an important role, with conflicts between the virtual and real environment causing conflicts (38). The effects of a subject's postural stability on cybersickness have conflicting evidence, with some studies claiming that more stable individuals are more prone to being affected whilst others claim the opposite (37). However, none of our subjects reported experiencing symptoms of postural instability, possibly due to there being visual cues in augmented reality which are not present in VR, thus reducing the risk of conflicting sensations.

The SSQ scale defines symptoms of concern, which are great enough to stop a person voluntarily using a device, at 15 and over (29). Other researchers found that severe symptoms, which they defined as SSQ scores over 30, were present in around 10% of users (28).. In our experiment, the median scores of participants did not exceed 15 in either group, with many participants describing negligible symptoms, which.is below the threshold of considered significant enough to prompt avoidance of further exposure (29).

A baseline SSQ score of 0 was assumed in the present methodology, following the approach originally proposed by Kennedy. However, the validity of this assumption has been questioned in the current literature, as it may artificially inflate post-exposure scores and consequently lead to an overestimation of cybersickness incidence, possibly impacting the internal validity (39). Conversely, the pre–post administration of the questionnaire has also been criticized, as completing the SSQ prior to exposure may draw participants' attention to potential symptoms by causing a heightened symptom awareness,inducing a nocebo effect, which could contribute overestimating cybersickness (40). However, the relatively low SSQ scores observed in this study suggest that such skew of cybersickness is unlikely to have substantially influenced our results

We found no correlation between age and cybersickness, neither positive, as suggested by Arns, nor negative, as reported by Mousavi (41, 42). We also found no correlation between gender and cybersickness, presumably because the field of view (FOV) of the head-mounted display (HMD) was limited to 110°, which is considerably narrower than the typical peripheral FOV in women (155°) and men (160°) (43). Reduced FOV has been shown to have a protective effect against cybersickness (28). Additionally, our results suggest that there was no effect related to the faster reaction times to peripheral visual stimuli observed in women compared to men, despite the fact that enhanced peripheral visual stimulation is known to significantly increase the illusion of self-motion, or vection (44, 45).

We did not believe that the fact that the HMD had an interpupillary distance (IPD) range of 55–75 mm, which does not fully accommodate individuals, typically women, with IPDs below 55 mm, would be relevant (43). While IPD mismatch has been shown to be positively correlated with increased Simulator Sickness Questionnaire (SSQ) scores, this limitation did not appear to contribute to gender differences in cybersickness in our study (46). Our analyses found a slight correlation between the time wearing the headset and symptoms. This would be in accordance with previous research, which suggests that time spent in AR may correlate with developing symptoms (47, 48). In alternative explanation is that this may be due to participants taking longer due to experiencing symptoms having more trouble completing the tasks given.

The relatively mild symptoms felt by participants may also be explained by the use of what could be considered, despite the affordable price, a “high-end” device according to some researchers (49). The resolution, refresh rate, field of view, and peak angular pixel density of the Quest 3 are among the highest actually available (24). In their study, it was found that higher-end devices, such as the Quest 3 and Apple's Vision Pro (Apple Inc, Cupertino, California, United States) were less likely to cause symptoms compared to lower-end devices that used smartphones as a display device when completing simple tasks (49). Furthermore, Wang recommends using refresh rates of 120 Hz or higher to reduce cybersickness, especially when visual attention to object details is critical to the task (50).

The use of AR can also theoretically bring potential ergonomic benefits by reducing the need for neck rotation and improving the user's line of sight by allowing the placement of the screen in a comfortable and practical area. In the HMD, one can have up to three virtual computer screens streamed directly from physical monitors, offering extensive possibilities that cannot be achieved through simple structural arrangements in the room (51). This consideration is all the more important due to the realization that most sonographers are suffering from painful work-related musculoskeletal disorders (6). Due to the prevalence of the problem, some countries include ergonomics as part of their sonography training, though recent studies show that these practices are challenging to put into place in real-world scenarios (52). In a large-scale study of sonographers it was found that being able to adjust the chair and keyboard were both associated with less pain, suggesting that positioning is a major factor in reducing musculoskeletal pain (53). With our setup which allows the user to position screen, as well as a virtual keyboard and settings menu, wherever they wish. Furthermore, the ability to use the in-built microphone to use dictation can eliminate the need for typing to label the images which are being taken. A relevant factor was the eye strain experienced by sonographers, especially those with sight-related issues (53). This is another factor where the ability to move the screen, to reposition and pin it within the environment, to bring it closer as well as to adjust the size to make it as large or as small as the user wants may play a significant role in reducing eye strain by allowing the user the flexibility to establish ergonomically appropriate working configuration

### Abdominal ultrasound

One group performed an experiment similar to our own using portable ultrasound probes and AR headset (54). However, unlike our experiment, they relied on custom hardware and software from MediView (MediView XR Inc, Cleveland, OH), which were the sponsors of their study, rather than on repurposed commercially accessible hardware and software. They also required a lengthy teaching session by the manufacturers to learn to use the material and required practice, whereas in our study the participants learnt how to use the system and completed the exams within the same short session. This difference may have been due to the custom software used in their experiment, which was criticized as being difficult to use, whereas since we made use of the familiar app and Meta's in-built software our participants had little trouble adapting, which is shown by the limited time they required to complete their exams (54). They also faced challenges due to requiring well-lit rooms, which interfered with their equipment (54). Using the Quest 3, however, we experienced no such problems as the glasses are designed for working in well-lit areas so that the external cameras are able to better record the environment, which is replicated on the screens, making them ideal for well-lit hospital settings.

### Vascular access procedures

It is common for medical institutions to provide access to ultrasound machines to facilitate vascular access procedures (16). When performing these procedures the ultrasound machine is often placed in close proximity to the practitioner, which can result in it being positioned at an awkward angle (16). This will require the practitioner to repeatedly turn their head and shift their focus back and forth between the site of the procedure and the ultrasound screen, which can result in loss of focus for less experienced practitioners (16).

Previous research has shown that when performing vascular access procedures using Google Glasses, practitioners spent more time looking at the augmented reality screen than when using a standard ultrasound monitor (16). This was surmised to be due to the easier access to the images having an encouraging effect on the practitioners, making them more aware of the needle's position and checking more regularly than they would otherwise do (16). Other researchers found they were able to achieve faster vascular access using a monocular head-mounted display (HMD) compared to standard procedures (13). In our study, though we did not compare the time taken to perform the procedure using a standard ultrasound setup, the procedures were conducted in a time-frame that is considered normal for the institution with a median time of around 12 min for a simulated abdominal exam and 7 min for a venipuncture. Notably, these times included the AR headset setup time and the time taken for the technicians to familiarize themselves with the AR equipment, which they were all using for the first time.

Another experiment comparing the time taken to perform a simulated nerve block using a standard ultrasound setup or one paired with a binocular HMD found that those using a HMD were able to perform the procedure significantly faster, whilst decreasing probe and head movements (55).

As well our first needle pass success rate of 87% was comparable to that reported in previous AR studies, which ranged from 83% to 90% (56).

### Practical limitations and considerations

Whilst performing this experiment, we encountered some practical limitations when using the AR equipment. One major limitation was the battery life of the headset, which usually lasted around 1.5 h, instead of the advertised 2.2 h. This may be explained in part by the headset having been purchased 3 years prior and thus suffering from usual battery degradation, though it is more likely due to the ultrasound probe being powered by the headset and thus draining the battery. In the future this could be overcome by external battery packs. A second limitation we encountered was a sudden drop-off in the capacity for the system to perform hand-tracking once the battery dropped to 30%. We could not explain this phenomenon but assumed it may be due to a battery-saving feature. A third limitation is that, due to the rapidity in which most exams were performed, it may have been too fast for significant cybersickness symptoms to occur. A fourth limitation is that, although in agreement with the original research protocol of Kennedy et al, no baseline SSQ scores were collected. Furthermore, as this was a single exam it may not be a correct simulation for the possible cumulative effects over an entire workday and other discomforts due to factors such as headset weight.

### Methodological limitations

Our experiment also suffers from some methodological limitations. One limitation is the lack of control group as we did not directly compare the subjects with a standard ultrasound setup. Secondly, our group size was relatively small, numbering only 52 divided into two groups. Thirdly, our subjects were only exposed for a short amount of time as the procedures were completed under 15 min each time. This represents a difference from the clinical reality of sonographers often working for many hours at time, which may compound the effects of cybersickness. The SSQ scale used is also a limitation, as it is known for over-estimating symptoms and providing a high score when even a few “slight” ratings are given. This scale is also limited by the time when the score is recorded and the lack of intervals between the measurements on the scale. SSQ remains the method of choice to measure cybersickness due to its widespread use, despite its known problem of over-estimating the score (28, 29).

As our research did not specifically focus on the ergonomic benefits that AR ultrasound may have over traditional techniques, this may prove a worthy avenue for future research. We also believe that future studies would benefit from SSQ scores being performed before the procedure, as well as the addition of a control group allowing for better focus on changes linked to the intervention. Another useful research avenue would be to formally assess the ergonomic advantages by surveys, EMG monitoring and posture tracking, amongst other potential techniques.

## Conclusion

This study provides preliminary feasibility data that consumer-grade AR head-mounted displays can be used to perform ultrasound procedures with minimal cybersickness and high task completion feasibility. Participants tolerated AR well and rapidly adapted to the interface, completing both abdominal and venous cannulation tasks without difficulty, though it should be noted that the power of this study was low. These findings support the practicality of integrating pass-through AR into medical imaging workflows, where it can expand display flexibility and may theoretically serve as a tool for improving ergonomics. However, the lack of severe SSQ scores does not necessarily equate to what would be experienced when performing ultrasound exams using a standard workflow.

Future work should evaluate performance in real clinical environments, quantify ergonomic benefits, and assess longer-duration use, with the addition of having direct comparisons with traditional ultrasound setups. Comparative studies with conventional ultrasound workflows and investigations of how AR interfaces impact diagnostic accuracy and training efficiency will be essential for guiding broader clinical adoption.

## Data Availability

The raw data supporting the conclusions of this article will be made available by the authors, without undue reservation.
